# A custom library construction method for super-resolution ribosome profiling in Arabidopsis

**DOI:** 10.1186/s13007-022-00947-2

**Published:** 2022-10-04

**Authors:** Hsin-Yen Larry Wu, Polly Yingshan Hsu

**Affiliations:** Department of Biochemistry & Molecular Biology, Michigan State University, East Lansing, MI 48824 USA

**Keywords:** Ribosome profiling, Ribo-seq, Ribosome footprint, 3-nt periodicity, Translatome

## Abstract

**Background:**

Ribosome profiling, also known as Ribo-seq, is a powerful technique to study genome-wide mRNA translation. It reveals the precise positions and quantification of ribosomes on mRNAs through deep sequencing of ribosome footprints. We previously optimized the resolution of this technique in plants. However, several key reagents in our original method have been discontinued, and thus, there is an urgent need to establish an alternative protocol.

**Results:**

Here we describe a step-by-step protocol that combines our optimized ribosome footprinting in plants with available custom library construction methods established in yeast and bacteria. We tested this protocol in 7-day-old Arabidopsis seedlings and evaluated the quality of the sequencing data regarding ribosome footprint length, mapped genomic features, and the periodic properties corresponding to actively translating ribosomes through open resource bioinformatic tools. We successfully generated high-quality Ribo-seq data comparable with our original method.

**Conclusions:**

We established a custom library construction method for super-resolution Ribo-seq in Arabidopsis. The experimental protocol and bioinformatic pipeline should be readily applicable to other plant tissues and species.

**Supplementary Information:**

The online version contains supplementary material available at 10.1186/s13007-022-00947-2.

## Background

Ribosome profiling (a.k.a. Ribo-seq) is a cutting-edge technique to study genome-wide mRNA translation through the deep sequencing of ribosome footprints (RFs) [1]. It maps and quantifies ribosome occupancy on mRNA, which enables the identification of coding regions and the accurate quantification of translation efficiency [2]. We previously optimized the Ribo-seq method in Arabidopsis and tomato [3–5] to obtain precise RFs with strong 3-nucleotide periodicity, a feature displayed by actively translating ribosomes and a benchmark of high-quality Ribo-seq [1, 2]. This strong 3-nt periodicity allowed us to confidently define numerous unannotated translation events and to study their underlying regulation across plants [3–5].

**Fig. 1:**
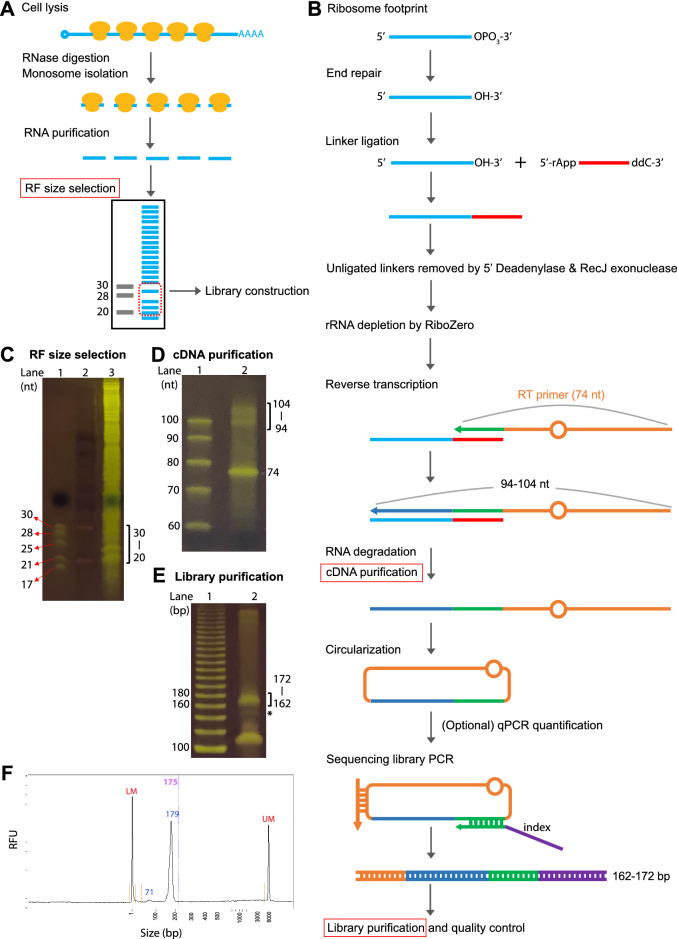
Ribo-seq workflow and representative gel images. **A** Ribo-seq overview: a Ribo-seq experiment consists of ribosome footprinting and sequencing library construction. **B** Workflow for custom Ribo-seq library construction. After size selection, ribosome footprints undergo end repair, ligation to a linker, removal of excess linkers, rRNA depletion, reverse transcription, cDNA purification, cDNA circularization, library PCR, and library purification. Among these steps, three (size selection of ribosome footprints, cDNA purification, and library purification) involve gel purifications and are highlighted by red boxes. For 20–30-nt ribosome footprints, the expected cDNA length would be 94–104 nt, and the expected library size would be 162–172 bp. **C** Size selection of ribosome footprints using a 15% TBE-urea gel. Lane 1: 30- and 28-nt marker (from the discontinued illumina Ribo-seq kit) and 25-, 21-, 17-nt marker (NEB microRNA marker). Lane 2: DynaMarker Prestain Marker for Small RNA Plus (Diagnocine). Lane 3: ribosome footprint sample. In this study, gel slices corresponding to the 20–30-nt range (marked by the bracket) were excised. **D** cDNA purification using a 10% TBE-urea gel. Lane 1: ssDNA ladder. Lane 2: cDNA sample. The bracket marks the expected cDNA length (94–104 nt), and the 74-nt band corresponds to the unused RT primer. **E** The resulting library after amplification with 11 cycles of PCR and resolved on an 8% TBE gel. Lane 1: 20-bp ladder. Lane 2: the library product; the bracket marks the expected library size (162–172 bp). The asterisk marks the product from an unextended RT primer, which should be avoided. **F** Fragment Analyzer profile showing enrichment of the expected library size. LM and UM are the vendor’s internal size markers

There are two phases in Ribo-seq experiments: ribosome footprinting and sequencing library construction. Ribosome footprinting involves (1) lysate preparation, (2) ribonuclease digestion, (3) monosome isolation and RNA purification, and (4) RF size selection. In addition, rRNA depletion is commonly deployed to eliminate abundant rRNA contaminants before or during library construction (Fig. 1A).

Important considerations in the major steps of ribosome footprinting include: (1) lysate preparation: it is critical to immobilize ribosomes on mRNAs to reflect their in vivo translational status. In eukaryotes, immobilization is typically achieved via freezing with liquid nitrogen and lysing the cells in the presence of the translation inhibitors cycloheximide (for cytosolic ribosomes) and chloramphenicol (for plastid and mitochondrial ribosomes). (2) Ribonuclease digestion: this step is the most critical for determining the nucleotide resolution of Ribo-seq. RNase I digestion may be performed without pre-purifying polysomes [3, 5, 6]. Plant materials with fewer active ribosomes, such as adult leaves, may require prior polysome enrichment [7]. Adjusting the pH and ionic strength of the lysis buffer and titrating the amount of RNase I help maximize the nucleotide resolution [3, 4]. (3) Monosome isolation: size exclusion columns, sucrose cushions, or sucrose gradients can be used to purify monosomes, but the latter two require ultracentrifugation and specialized equipment. (4) RF size selection: this has a profound effect on the Ribo-seq reads obtained and should be carefully evaluated depending on the study objective. Our previous and other studies select footprints of 28–30 nt [3, 5, 6], which is the major length of cytosolic RFs. Some studies isolate a wider range of footprint lengths (e.g., 20–40 nt) to capture plastid ribosomes (~ 32 nt), as well as a subset of cytosolic ribosomes (21–22 nt) [8]. (5) rRNA depletion: without rRNA depletion, around 90% of the resulting Ribo-seq reads would comprise contaminant rRNA fragments [9]. Two common approaches to deplete rRNAs are (i) using a commercial illumina RiboZero kit, and (ii) subtractive hybridization of biotinylated oligos customized to specific tissues and library preparations, which requires a pilot Ribo-seq experiment to identify the abundant contaminant sequences.

We previously used illumina Ribo-seq kit and standalone RiboZero in our Ribo-seq protocol [3–5]. The discontinuation of these reagents prompted us to test an alternative library construction method. Here, we describe a protocol that combines our optimized ribosome footprinting method in Arabidopsis [3, 5] with custom library construction methods in yeast and bacteria [10, 11]. We applied this protocol to 7-day-old Arabidopsis seedlings and assessed the quality of the resulting libraries using open resource bioinformatic tools. The step-by-step protocol with detailed reagent information, as well as the analysis code and example dataset description, are provided in Additional files 1, 2, 3, 4 and https://github.com/hsinyenwu/Riboseq_protocol_2022.

## Results and discussion

### Construction of Ribo-seq libraries using a custom method

Figure 1 shows the workflow of our new Ribo-seq protocol. We followed our optimized ribosome footprinting method, which yields strong 3-nt periodicity for open reading frame (ORF) identification [3, 5]. After isolating monosomes and purifying RNA, a distinct band corresponding to ~ 28 nt could be observed in a polyacrylamide gel (Fig. 1C and Additional file 5: Fig. S1). Instead of selecting 28–30-nt RFs, in this study, we selected 20–30-nt RFs to capture both 21–22-nt and 28-nt RFs. Our new library construction workflow is summarized in Fig. 1B. Briefly, the RFs are end repaired to allow their 3′ end to ligate to a pre-adenylated linker. Unligated linkers are removed by 5′ deadenylase and RecJ (an ssDNA exonuclease acting in the 5′-to-3′ direction). Following rRNA depletion by RiboZero (available as a component in the illumina TruSeq RNA-seq kit for plants), a reverse transcription (RT) primer that complements the linker enables RF cDNA synthesis. After the RNA is degraded and the cDNA is gel-purified from unutilized RT primers (Fig. 1D), the resulting cDNA is circularized. qPCR is performed as recommended [10] to quantify the circularized cDNA and estimate the amount of input and number of PCR cycles needed next. Finally, library construction PCR is performed, where indexes and illumina sequencing sequences are incorporated. The libraries are gel-purified and evaluated with Fragment Analyzer before sequencing (Fig. 1E, F). We found the established guideline for qPCR quantification and cycles of library PCR [10] worked well for our Arabidopsis samples (Additional file 5: Fig. S2) and yielded sufficient library DNA for sequencing.

### Evaluation of high-quality Ribo-seq data

**Fig. 2:**
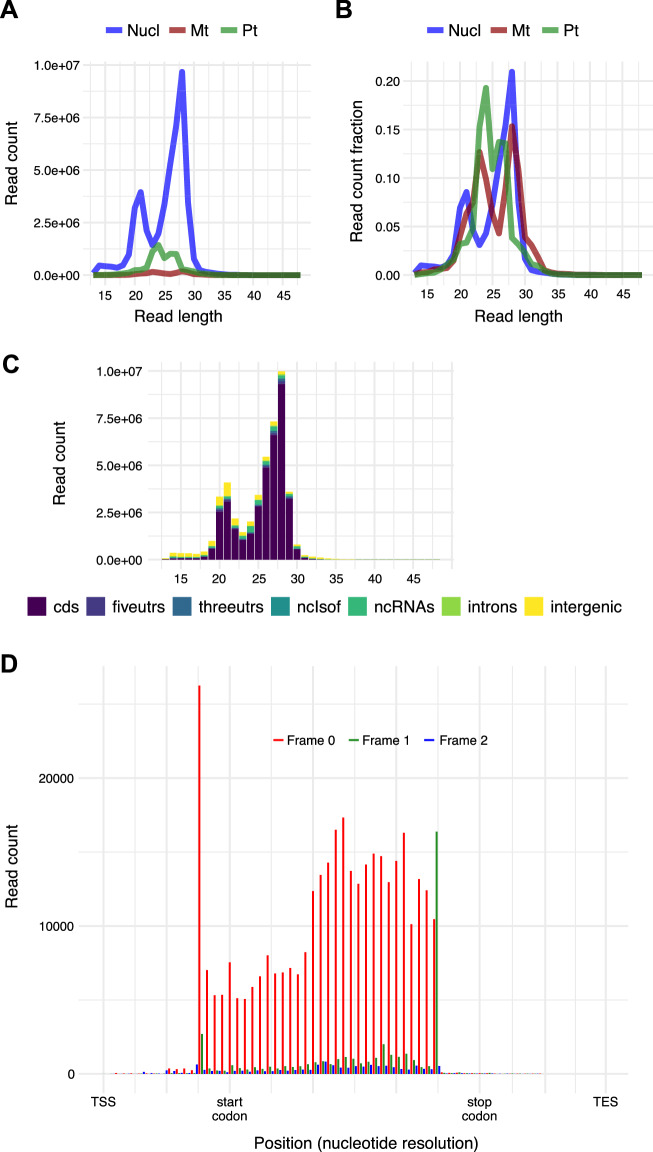
Assessments of Ribo-seq data. **A**, **B** Read length distribution of nucleus- (Nucl), mitochondria- (Mt) and plastid- (Pt) encoded transcripts displayed based on read counts (**A**) or fractions (**B**). **C** Genomic features of ribosome footprints grouped by read length. Different genomic features are shown with different colors. Ribosome footprints that mapped to nucleus-encoded genes are presented. **D** Metaplot of protein-coding transcripts showing strong 3-nt periodicity and high enrichment within expected coding regions. Reads mapped to the three reading frames are shown in red, green and blue. The ribosome footprint position is shown by the first nucleotide of the footprint; thus, the first peak is 12 nt upstream of the start codon, consistent with our previous datasets [3–5]

The sequencing data were first pre-processed, and common contaminant sequences (Additional file 3) from rRNAs/tRNAs/snRNAs/snoRNAs and a few overrepresented non-coding RNAs were removed. Overall, we obtained similar rRNA depletion results (Additional file 5: Fig. S3, ~ 30% rRNA contamination remained) compared to our previous dataset [3] (19–41% rRNA contamination remained). After mapping the Ribo-seq reads to individual transcripts, the three technical replicates showed excellent correlations (Additional file 5: Fig. S4, Pearson correlation coefficient r = 0.99). We pooled all three replicates and used Ribo-seQC [12] to assess the data quality. As expected, for the nucleus-encoded genes, the major RF length was 28 nt (Fig. 2A, B). A minor peak at 21 nt, which represents the ribosomes in the rotated conformation [13], was also observed (Fig. 2A, B). For the plastid-encoded genes, 23–27 nt RFs were enriched (Fig. 2A, B). Consistent with our expectation, most of the RFs mapped to coding sequences across all RF lengths (Fig. 2C). In the metaplot (i.e., global analysis of all Ribo-seq reads, where RFs map near the start codons, the middle of coding sequence, and stop codons of annotated protein-coding genes were shown) (Fig. 2D), strong 3-nt periodicity was present within the coding sequences as 91.03% of reads were mapped to the expected reading frame shown in red. Moreover, sparse reads were present within the 5′ and 3′ UTRs, as expected (Fig. 2D). The strong 3-nt periodicity was also comparable to our previous data in Arabidopsis root and shoot [3] (Additional file 5: Fig. S5). Overall, our new protocol yields high-quality Ribo-seq data considering RF length distribution, genomic location distribution, and the fraction of RFs enriched in the expected reading frame.

### Considerations for future Ribo-seq experiments

Compared to our previous narrow RF size selection (28–30 nt), selecting a wider range of sizes (20–30 nt) in this study allowed us to obtain 21-nt RFs in the rotated conformation [13]. The tradeoffs are that we had more contaminating sequences, especially at 25 nt (Additional file 5: Figs. S6 and S7), and shorter RFs are expected to have a lower mapping rate. Depending on the purpose of the study, one might prefer to select different RF sizes. For studies focusing on cytosolic translation, one might omit chloramphenicol in the lysis buffer. If desired, one could consider including a unique molecular identifier, i.e., degenerate sequences, at the 5′ end of the linker and/or the 5′ end of the RT primer to reduce and correct bias introduced by ligation and PCR amplification [6, 10].

## Conclusions

In summary, we have implemented a custom library construction protocol for our optimized ribosome footprinting method in Arabidopsis. The experimental protocol and bioinformatic pipeline should readily apply to other plant tissues and species.

## Methods

Below, we describe the key steps in a ribosome profiling experiment and subsequent data analysis. A step-by-step Ribo-seq protocol and detailed reagent information are provided in Additional file 1. The code for the data analysis is provided in Additional file 2. Common contaminating sequences are provided in Additional file 3. A detailed description of data analysis, including an example dataset, is provided at https://github.com/hsinyenwu/Riboseq_protocol_2022 and Additional file 4. The GitHub page also contains links to other Ribo-seq analysis tutorials that readers might find helpful.

### Plant materials

Seven-day-old Arabidopsis seedlings were grown hydroponically in sterile liquid media (2.37 g/L LS pH 5.7, 1% sucrose, 0.5 g/L MES) with shaking at 85 rpm under a 16-h light (~ 55 μmol m^−2^ s^−1^ from cool white fluorescent bulbs) and 8-h dark cycle at 22 °C. Whole seedlings were harvested at Zeitgeber time 4 (4 h after lights on). After removing excess media with paper towels, the plant materials were immediately placed in foil, flash-frozen in liquid nitrogen, and used for lysate preparation.

### Lysate preparation

The lysates and ribosome footprints were prepared according to our previous methods [3, 5]. Ice-cold lysis buffer (100 mM Tris–HCl [pH 8], 40 mM KCl, 20 mM MgCl2, 2% [v/v] polyoxyethylene [10] tridecyl ether [Sigma, P2393], 1% [w/v] sodium deoxycholate [Sigma, D6750], 1 mM dithiothreitol, 100 µg/mL cycloheximide [Sigma, C4859], 100 µg/mL chloramphenicol [Sigma R4408], and 10 units/mL DNase I [Lucigen, D9905K]) was prepared and aliquoted in 5-mL centrifuge tubes. After grinding the frozen tissue to a fine powder with a mortar and a pestle, the powder was swept into the aliquoted lysis buffer (0.1 g per 400 µL lysis buffer) and immediately resuspended by vortexing. The lysates were mixed at 4 °C for 10 min with shaking, then spun at 5000×*g* for 3 min at 4 °C. The supernatant was transferred to new tubes and spun at 20,000×*g* for 10 min at 4 °C. The supernatant was transferred to new tubes again, and 200-µL (for ribosome footprint preparation) and 50-µL (for total RNA extraction) aliquots were made. The aliquots were frozen in liquid nitrogen and saved at -80 °C until further processing.

### Ribosome footprinting, monosome isolation and size selection

The 200-µL lysate aliquots above were processed to generate ribosome footprints. The RNA concentration was determined via Qubit RNA high-sensitivity assay (Thermo Fisher Scientific, Q32855) using tenfold diluted lysate. RNase I (Lucigen N6901K, 50 U/per 40 µg RNA) was added to the lysates, and the reactions were mixed on a nutator at room temperature for 1 h. The reactions were terminated by placing the samples on ice and adding 15 µL SUPERase-In (Thermo Fisher Scientific AM2696). Monosomes were isolated by applying each 100 µL of digested lysate onto one size exclusion column (illustra MicoSpin S-400 HR, GE Healthcare 27-5140-01), which was equilibrated with 3 mL of polysome buffer (100 mM Tris–HCl [pH 8], 40 mM KCl, 20 mM MgCl_2_) in advance. Then, 10 µL of 10% SDS was added to the elute, and RNA > 17 nt was isolated using an RNA Clean & Concentrator kit (Zymo Research R1015). The purified RNA was separated via 15% (w/v) TBE-urea PAGE (Thermo Fisher Scientific; EC68852BOX), and gel slices corresponding to 20–30 nt were excised. Ribosome footprints were recovered and used for library construction.

### Ribo-seq library construction

The library construction method was modified from two methods [10, 11]. The ribosome footprints were repaired via T4 PNK (NEB M0201S) in the absence of ATP and ligated to a universal miRNA cloning linker (NEB S1315S) with T4 RNA Ligase 2 truncated K227Q (NEB M0351S). The excess linkers were removed with 5′ deadenylase (NEB M0331S) and RecJf (NEB M0264S). After purifying the ligation products using an Oligo Clean & Concentrator kit (Zymo D4061), rRNA depletion was performed using RiboZero, which is included in the TruSeq Stranded Total RNA Library Prep Plant kit (illumina 20020610), and the ligated ribosome footprints were purified again with an Oligo Clean & Concentrator kit. Next, reverse transcription was carried out with ProtoScript II (NEB M0368L) and a reverse transcription primer whose sequence at the 3’ end was complementary to the linker. The RNA was degraded by treating the sample with NaOH at a final concentration of ~ 0.1 M. The cDNA was purified again with an Oligo Clean & Concentrator kit and separated via 10% (w/v) TBE-urea PAGE (Thermo Fisher Scientific EC68752BOX). Then, cDNA of the expected size (94–104 nt) was selected, recovered, and circularized using CircLigase (Lucigen CL4111K).

### qPCR quantification of circularized cDNA

The circularized cDNA was quantified via qPCR using Luna universal qPCR master mix (NEB M3003S). A synthesized oligo was used as a positive control and to establish the standard curve for quantification following the guideline described in [10]. The primer and oligo sequences are listed in Additional file 1.

### Library PCR amplification, purification, and sequencing

Eleven-cycle library PCR with indexed primers was performed using Phusion high-fidelity PCR master mix (NEB M0531S) following the recommendations based on the qPCR quantification [10]. The PCR products were separated on an 8% TBE gel (Thermo Fisher Scientific EC62152BOX), and the library of the expected size (162–172 bp) was selected and recovered. The size of the recovered libraries was evaluated using Fragment Analyzer (Agilent). The libraries were quantified via Qubit dsDNA HS assay (Thermo Fisher Scientific Q32854) and pooled at equal molarity. Single-end 50-bp sequencing was performed in a HiSeq 4000. The raw sequencing data have been deposited in the NCBI Sequence Read Archive (SRA) under BioProject ID PRJNA854638.

### Data analysis

(The code used in this study is provided in Additional file 2 and at the GitHub link (https://github.com/hsinyenwu/Riboseq_protocol_2022).

- **Step 1** (Trim adaptors and remove low-quality sequences): the adaptor sequence (CTGTAGGCACCATCAAT) was first trimmed from the Ribo-seq reads with *fastx_clipper* (*fastx toolkit* v0.11.7, http://hannonlab.cshl.edu/fastx_toolkit/). For the *fastx_clipper* function, the -c, -n, -v, -Q33 options were used to discard unclipped reads, keep reads with unknown nucleotides, and provide verbose output, and the Q33 filter was used to remove low-quality reads.
- **Step 2** (Remove contaminating sequences with *Bowtie2*): we first built a *Bowtie2* (v. 2.3.4.1) [14] index with the *bowtie2-build* function for removing unwanted contaminating sequences such as those from rRNAs, tRNAs, snRNAs, and snoRNAs. We also used the same method to remove additional high-abundance non-coding RNAs (i.e., *AT3G06365* and *AT2G03875*). These contaminating sequences are listed in Additional file 3. We next used *Bowtie2* with seed length (-L option) 20 to extract Ribo-seq sequences that did not map to contaminating sequences.
- **Step 3** (Map Ribo-seq reads): we then created an index file for *STAR* aligner (v 6.2.0c) [15] against the Araport11 transcriptome and the TAIR10 genome using the following options: --runMode genomeGenerate, --sjdbOverhang 34.
  We mapped the remaining Ribo-seq reads from Step 2 with *STAR* aligner using the following options: --alignIntronMax 5000, --alignIntronMin 15, --outFilterMismatchNmax 1, --outFilterMultimapNmax 20, --outFilterType BySJout, --alignSJoverhangMin 8, --alignSJDBoverhangMin 2, --outSAMtype BAM SortedByCoordinate, --quantMode TranscriptomeSAM, --outSAMmultNmax 1, --outMultimapperOrder Random.
- **Step 4** (Conduct Ribo-seQC analysis): next, we used the Ribo-seQC package [12] to analyze the quality of the Ribo-seq reads. We first used the *export.2bit* function from the *rtracklayer* package [16] to create the 2bit file for the TAIR10 genome. We generated the annotation file for *Ribo-seQC* with the *prepare_annotation_files* function using the Araport11 gtf file and the genome 2bit file as inputs. The *Ribo-seQC* output was generated using the *RiboseQC_analysis* function with the *Ribo-seQC* annotation file and the bam file generated in Step 3.
- **Step 5** (Conduct Kallisto quantification for Ribo-seq data and correlation analysis): we used Kallisto [17] to create the index and quantify the three technical replicates. We then used the corrplot function from the corrplot library in R (v4.0.3) [18] to plot the correlation of the three replicates.
- **Step 6** (Calculate 3-nt periodicity): we used the output file (ending with bam_results_RiboseQC) from Ribo-seQC to calculate 3-nt periodicity in R. A total of 93 nucleotides (31 codons) were considered, including 33 nucleotides starting from the start codon, 33 nucleotides in the middle of the transcript, and 27 nucleotides from − 2 to − 10 codons upstream of the stop codon.

## Supplementary Information

**Additional file 1.** Step-by-step Ribo-seq protocol.

**Additional file 2.** Code used in this study.

**Additional file 3.** Contaminating sequences from rRNAs, tRNAs, snRNAs, snoRNAs and dominant noncoding RNAs.

**Additional file 4.** Markdown file of example data analysis.

**Additional file 5: Figure S1.** Precise ribonuclease digestion yields a clear band between 28 and 30 nt. **Figure S2.** qPCR quantification of circularized cDNAs and estimation of template volumes needed for library construction PCR. **Figure S3.** Comparison of rRNA contamination in our previous and current datasets. **Figure S4.** High correlations among three technical replicates of Ribo-seq data. **Figure S5.** Comparable strong 3-nt periodicity in our current data. **Figure S6.** Abundant contamination sequences present at 25 nt. **Figure S7.** Mapping and contamination statistics. Contaminant sequences considered include rRNAs/tRNAs/snRNAs/snoRNAs and overrepresented non-coding RNAs. Their sequences are listed in Additional file 3.

## Data Availability

The raw sequencing data generated in this study have been submitted to the NCBI Sequence Read Archive (SRA) under BioProject ID PRJNA854638. The data analysis code and an example dataset are available at https://github.com/hsinyenwu/Riboseq_protocol_2022.
