# Supplementary material for: A custom library construction method for super-resolution ribosome profiling in Arabidopsis
Source: Plant Methods. 2022 Oct 4;18:115. doi: 10.1186/s13007-022-00947-2 (PMC9531494; doi:10.1186/s13007-022-00947-2)
Supplement: Supplementary file 1 — Additional file 1. Step-by-step Ribo-seq protocol. [file 13007_2022_947_MOESM1_ESM.pdf]

ADDITIONAL FILE 1: Step-by-step Ribo-seq protocol

REAGENTS

Material	Manufacturer	Catalog No.
RNaseZap RNase Decontamination Solution (Optional)	Thermo Fisher Scientific	AM9780
Liquid nitrogen		
Mortar, 50 mL, 47 x 70 mm	VWR	89038-144
Pestle, 50 mL, 114 mm	VWR	89038-160
Nuclease-Free Water (not DEPC-Treated)	Thermo Fisher Scientific	4387936
Sodium deoxycholate	Sigma	D6750-100G
Polyoxyethylene (10) tridecyl ether	Sigma	P2393-100G
2 M KCl	Thermo Fisher Scientific	AM9640G
1 M $MgCl_2$	Thermo Fisher Scientific	AM9530G
1 M Tris pH 8.0	Thermo Fisher Scientific	AM9855G
10X TBE Buffer	Thermo Fisher Scientific	AM9863
Chloramphenicol, ready made solution	Sigma	R4408-10ML
Cycloheximide, ready made solution	Sigma	C4859-1ML
DL-dithiothreitol (DTT) solution, BioUltra	Sigma	43816-10ML
RNase-free DNase I	Lucigen	D9905K
RNase I, E. coli	Lucigen	N6901K
SUPERase-In	Thermo Fisher Scientific	AM2696
illustra MicroSpin S-400 HR Columns	GE Healthcare	27-5140-01
10% SDS solution	Thermo Fisher Scientific	AM9822
RNA Clean & Concentrator-5 no DNase I	Zymo	R1015
Oligo Clean & Concentrator	Zymo	D4061

DNA Clean & Concentrator-5 (Capped)	Zymo	D4013
5 M NaCl	Thermo Fisher Scientific	AM9760G
3M NaOAc pH 5.5	Thermo Fisher Scientific	AM9740
0.5 M EDTA pH 8.0	Thermo Fisher Scientific	AM9260G
Ethanol, 200 proof	Decon Labs	V1016
Isopropanol, molecular biology grade	Fisher	BP2618500
GlycoBlue (15 mg/mL)	Thermo Fisher	AM9515
15% TBE-urea gel 12 well	Thermo Fisher Scientific	EC68852BOX
10% TBE-urea gel 12 well	Thermo Fisher Scientific	EC68752BOX
8% TBE gel, 12 wells	Thermo Fisher Scientific	EC62152BOX
Disposable transfer pipette	Fisher	13-711-20
DynaMarker, Prestain Marker for Small RNA Plus	Diagnocine	DM253
miRNA marker (optional)	NEB	N2102S
20/100 Ladder	IDT	51-05-15-02
20 bp Ladder	Bayou Biolabs	L-100
Gel loading buffer II (Denaturing PAGE)	Thermo Fisher Scientific	AM8546G
Gel loading dye, purple (6X)	NEB	B7024S
SYBR Gold nucleic acid gel stain 500 µL	Thermo Fisher Scientific	S11494
TruSeq Stranded Total RNA Library Prep Plant	illumina	20020610
QUBIT RNA HS assay kit	Thermo Fisher Scientific	Q32855
QUBIT dsDNA HS assay	Thermo Fisher Scientific	Q32854
T4 polynucleotide kinase (PNK)	NEB	M0201S

Universal miRNA cloning linker	NEB	S1315S
T4 RNA Ligase 2 truncated K227Q	NEB	M0351S
5´ Deadenylase	NEB	M0331S
RecJf	NEB	M0264S
ProtoScript II	NEB	M0368L
dNTP (10mM each)	Thermo Fisher Scientific	R0191
Sodium hydroxide	Fisher	S318500
CircLigase	Lucigen	CL4111K
Luna universal qPCR master mix	NEB	M3003S
Phusion high-fidelity PCR master mix with HF buffer	NEB	M0531S
Olympus 5ml Centrifuge tube	Genesee	24-285S
Ultrafree-MC GV centrifugal filter	EMD Millipore	UFC30GV00
MAXYMum Recovery PCR tubes	VWR	22234_056
Non-sticky RNase-free tubes 1.5 mL	Thermo Fisher Scientific	50591363
10µL pipette filter tips, low binding, sterile	Genesee	24_401
20µL pipette filter tips, low binding, sterile	Genesee	24_404
200µL pipette filter tips, low binding, sterile	Genesee	24_412
1000µL pipette filter tips, low binding, sterile	Genesee	24_430
200 µL round gel tip, 0.58 mm	Genesee	14_101
MicroAmp fast optical 96-well reaction plate	Thermo Fisher Scientific	4346906
TempPlate RT qPCR sealing film	USA Scientific	2978-2100
0.2 mL PCR 8-tube strip with 8-cap strips	Genesee	24_705

EQUIPMENT

Equipment	Manufacturer	Catalog No.
Refrigerated centrifuge for 5 mL tubes and 1.5 mL tubes	Eppendorf	5430R
BD Clay Adams Nutator Mixer	VWR	15172-203
Qubit fluorometer	Thermo Fisher Scientific	Q33238
Mini gel tank	Thermo Fisher Scientific	A25977
Electrophoresis power supply	Fisher	FB300Q
DarkReader (Dark Field Transilluminator)	Clare Chemical Research	DR46B
T100 thermocycler	Bio-Rad	1861096
Magnetic Separation Stand, 12 position, 1.5 mL	Promega	Z5342
QuantStudio 3 Real-Time PCR System	Thermo Fisher Scientific	A28567

OLIGOS (all except the Universal miRNA cloning linker can be ordered from IDT)

- All oligos should be dissolved in nuclease-free water (NF-H2O) at the appropriate concentrations, aliquoted, and stored at -20°C.
- The linker sequence is shown in red, and the sequence complementary to the linker is shown in red and underlined.
- The forward library PCR primer sequence is shown in blue
- The shared sequence in the reverse qPCR primer and the indexed reversed library PCR primer is shown in green; its complementary sequence is shown in green and underlined

Oligo name	Scale & Purification	Sequence	Conc.
Universal miRNA cloning linker (5′ adenylated, 3′ blocked)	NEB S1513S	(5´) rAppCTGTAGGCACCATCAAT-NH2 (3´)	20 μM

Reverse transcription primer	100 nm, HPLC	/5Phos/AGATCGGAAGAGCGTCGTGTAGGGAAAGAGTGT/iSp18/CAAGCAGAAGACGGCATACGAGATATTGATGGTGCCTACAG	1.25 µM
Forward library PCR primer	25 nm, standard desalt	5'-CAAGCAGAAGACGGCATACGA-3'	10 µM
Reverse qPCR primer	25 nm, standard desalt	5'-ACACTCTTTCCCTACACGACG-3'	10 µM
Positive control for qPCR (100 nt including **26-nt-synthetic/hypothetical RF sequence)**	4 nm ultramer, standard desalt	5'CAAGCAGAAGACGGCATACGAGATATTGATGGTGCCTACAG**TCGCATTACCCTGTTATCCCTAACAT**AGATCGGAAGAGCGTCGTGTAGGGAAAGAGTGT3'	10 µM
Indexed reverse library PCR primer 1	4 nm ultramer, standard desalt	5’AATGATACGGCGACCACCGAGATCTACACGATCGGAAGAGCACACGTCTGAACTCCAGTCAC**ATCACG**ACACTCTTTCCCTACAC3’	10 µM
Indexed reverse library PCR primer 2	4 nm ultramer, standard desalt	5’AATGATACGGCGACCACCGAGATCTACACGATCGGAAGAGCACACGTCTGAACTCCAGTCAC**CGATGT**ACACTCTTTCCCTACAC3’	10 µM
Indexed reverse library PCR primer 3	4 nm ultramer, standard desalt	5’AATGATACGGCGACCACCGAGATCTACACGATCGGAAGAGCACACGTCTGAACTCCAGTCAC**TTAGGC**ACACTCTTTCCCTACAC3’	10 µM
Indexed reverse library PCR primer 4	4 nm ultramer, standard desalt	5’AATGATACGGCGACCACCGAGATCTACACGATCGGAAGAGCACACGTCTGAACTCCAGTCAC**TGACCA**ACACTCTTTCCCTACAC3’	10 µM
Indexed reverse library PCR primer 5	4 nm ultramer, standard desalt	5’AATGATACGGCGACCACCGAGATCTACACGATCGGAAGAGCACACGTCTGAACTCCAGTCAC**ACAGTG**ACACTCTTTCCCTACAC3’	10 µM

Indexed reverse library PCR primer 6	4 nm ultramer, standard desalt	5'AATGATACGGCGACCACCGAGATCTACACGATCGGAAGAGCACACGTCTGAACTCCAGTCAC**GCCAAT**ACACTCTTTCCCTACAC3'	10 µM
Indexed reverse library PCR primer 7	4 nm ultramer, standard desalt	5'AATGATACGGCGACCACCGAGATCTACACGATCGGAAGAGCACACGTCTGAACTCCAGTCAC**CAGATC**ACACTCTTTCCCTACAC3'	10 µM
Indexed reverse library PCR primer 8	4 nm ultramer, standard desalt	5'AATGATACGGCGACCACCGAGATCTACACGATCGGAAGAGCACACGTCTGAACTCCAGTCAC**ACTTGA**ACACTCTTTCCCTACAC3'	10 µM
Indexed reverse library PCR primer 9	4 nm ultramer, standard desalt	5'AATGATACGGCGACCACCGAGATCTACACGATCGGAAGAGCACACGTCTGAACTCCAGTCAC**GATCAG**ACACTCTTTCCCTACAC3'	10 µM
Indexed reverse library PCR primer 10	4 nm ultramer, standard desalt	5'AATGATACGGCGACCACCGAGATCTACACGATCGGAAGAGCACACGTCTGAACTCCAGTCAC**TAGCTT**ACACTCTTTCCCTACAC3'	10 µM
Indexed reverse library PCR primer 11	4 nm ultramer, standard desalt	5'AATGATACGGCGACCACCGAGATCTACACGATCGGAAGAGCACACGTCTGAACTCCAGTCAC**GGCTAC**ACACTCTTTCCCTACAC3'	10 µM
Indexed reverse library PCR primer 12	4 nm ultramer, standard desalt	5'AATGATACGGCGACCACCGAGATCTACACGATCGGAAGAGCACACGTCTGAACTCCAGTCAC**CTTGTA**ACACTCTTTCCCTACAC3'	10 µM

REAGENT SETUP:

- All reagents and supplies should be handled with care to ensure an RNase-free environment.
- Nuclease-free water is abbreviated “NF-H2O”

1. Cycloheximide stock solution (100 mg/mL), aliquot and store at -80°C
2. Chloramphenicol stock solution (100 mg/mL), aliquot and store at -80°C
3. 20% (v/v) PTE (polyoxyethylene (10) tridecyl ether): dispense 10 mL in a 50-mL sterile centrifuge tube, dissolve in NF-H2O and adjust to a final volume of 50 mL, store at room temperature
4. 10% (w/v) sodium deoxycholate: weigh 5 g in a sterile 50-mL centrifuge tube, dissolve in NF-H2O and adjust to a final volume of 50 mL, store at room temperature
5. 1 M DTT, aliquot and store at -80°C
6. 1 M NaOH: dissolve 0.04 g in 1 mL NF-H2O, store at room temperature
7. RNA extraction buffer, store at room temperature

	50 mL	Final concentration
NF-H2O	43.65	-
3M NaOAc, pH5.5	5	300 mM
10% SDS	1.25	0.25%
0.5M EDTA	0.1	1 mM

8. DNA extraction buffer, store at room temperature

	50 mL	Final concentration
NF-H2O	46.4	-
5M NaCl	3	300 mM
1M Tris, pH 8	0.5	10 mM
0.5M EDTA	0.1	1 mM

9. NEB Universal miRNA Cloning Linker:
    - Resuspend in 43 μL NF-H2O (final 20 μM)
    - Aliquot and store at -20°C
10. Tris (10 mM, pH8): dilute 1 M Tris pH 8 stock with NF-H2O, store at room temperature

PROCEDURES:

- All heating steps are performed in a thermocycler
- Starting with step 27, use low-bind (or non-sticky) 1.5-mL tubes and PCR tubes

A. Preparation of plant lysates

1. Make lysis buffer (add cycloheximide, chloramphenicol, DNase I, and DDT fresh); chill the buffer on ice:

	1 mL		50 mL		Final conc.
Nuclease-free water (NF-H2O)	647	μL	32.35	mL	-
1M Tris-HCl, pH 8	100	μL	5	mL	100 mM
20% Polyoxyethylene (10) tridecyl ether **(before Sodium deoxycholate)**	100	μL	5	mL	2%
10% Sodium deoxycholate	100	μL	5	mL	1%
2M KCl	20	μL	1	mL	40 mM
1M MgCl2	20	μL	1	mL	20 mM
1M DTT	1	μL	50	μL	1 mM
100 mg/mL Cycloheximide	1	μL	50	μL	100 μg/mL
100 mg/mL Chloramphenicol	1	μL	50	μL	100 μg/mL
DNase I (1U/μL)	10	μL	500	μL	10 U/mL

2. Aliquot the lysis buffer into 5 mL centrifuge tubes: 400 μL lysis buffer is needed for 0.1 g of whole-seedling Arabidopsis samples.
3. Collect samples in aluminum foil and freeze in liquid nitrogen immediately.
4. (Chill the centrifuges) Tare the 5 mL tube with lysis buffer on a scale first. Then, grind the samples using a chilled mortar and pestle with liquid nitrogen. Sweep the ground tissue into the buffer and weigh. Work quickly to prevent the tissue from thawing. Add more lysis buffer if needed to keep the tissue: buffer ratio consistent. Vortex to thoroughly resuspend the tissue. Leave the sample on ice while processing the other samples.
5. Vortex all samples again. Shake the samples at 4°C (on ice or in a cold room) for 10 min.
6. Spin shoot samples at 5,000 × g at 4°C for 3 min. During the spin, prepare new 1.5-mL tubes for the next step and make holes in the ice with a spare 5-mL tube to avoid disturbing the tissue debris after the spin.

7. Transfer the supernatant to chilled 1.5-mL tubes. Centrifuge at 20,000 × g at 4°C for 10 min. During the spin, prepare new 1.5-mL tubes for the next step and make holes in the ice with a spare 1.5-mL tube to avoid disturbing the tissue debris after the spin.
8. Transfer the supernatant to new chilled 1.5-mL tubes.
9. (Optional) Quantify the RNA concentration with a Qubit RNA HS assay using 10X diluted lysate.
10. (Optional) Adjust the samples to the same RNA concentration using lysis buffer.
11. Make aliquots of 200 µL (for ribosome footprint samples) and 50 µL (for RNA samples). Flash freeze the aliquots with liquid nitrogen and store at -80°C.

B. RNase I digestion & isolation of ribosome-protected fragments

12. (Do this during one of the buffer steps below if not done already) Quantify RNA concentration with a Qubit RNA HS assay using 10X diluted lysate.
13. Prepare size exclusion columns (SECs) and SEC buffer.
    - Vortex and invert the SECs several times to resuspend the resin. Remove both ends of the columns and place them on a rack to allow gravity flow.
    - Prepare 3 mL SEC buffer for each column; 1 column is used for 100 µL of lysate.

	1 mL		50 mL	
NF-H2O	860	µL	43	mL
1M Tris-HCl, pH 8	100	µL	5	mL
2M KCl	20	µL	1	mL
1M MgCl2	20	µL	1	mL

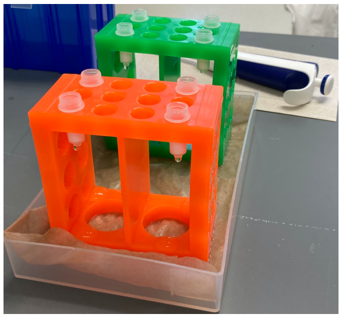
Set up SECs on a rack.

    - Resuspend the resin on the column cap using 0.5 mL SEC buffer by pipetting and adding it to the column. Initiate the flow with a gloved finger if necessary.
    - Add 0.5 mL SEC buffer every 15-20 min; after adding the buffer 3 times, set up the digestion (i.e., start step 14 right after adding the buffer for the 4th time).
    - After equilibrating the column with 3 mL of SEC buffer, tight the screw cap on all the way and then turn it ¼ turn to loosen the cap. Spin at 600 × g for 4 min before the digestion is almost done.
14. Add 50 units of RNase I per 40 µg RNA for each sample (200 µL lysate). Briefly vortex. Gently mix on a nutator for 1 hour.

15. Quickly move the samples to ice. Add 15 µL (per 200 µL lysate) of SUPERase-IN and mix.
16. Isolate monosomes by loading ~107 µL of digested lysate onto the center of the column. Spin at 600 × g for 2 min.
17. Add 10 µL of 10% SDS to each SEC elution.
18. Purify RNA > 17 nt with a modified Zymo RNA Clean & Concentrator-5 protocol
    - In step 1 of the Zymo protocol, use 290 µL RNA Binding Buffer
    - In step 2 of the Zymo protocol, use 655 µL EtOH
    - Repeat Zymo protocol step 3 to load the same sample if > 800 µL (we typically use 1 Zymo column to combine 2 SEC elutes of the same sample together; the RNA yield is within the binding capacity of the Zymo column 10 µg)
    - Continue with the purification according to the manufacturer's instructions
    - Elute with 11 µL 10 mM Tris pH 8
    - Combine 1 µL elution with 9 µL 10 mM Tris pH 8. This will be used for Qubit quantification and to check the digestion
    - The other ~10 µL elution will be used in Step 23.
19. Quantify the RNA concentration (use the 10X diluted sample above) using a Qubit RNA HS.
20. (Optional) Run 200 ng RNA (use the 10X diluted sample above) in a 15% TBE-urea gel to check the digestion
    - Prerun a 15% TBE-urea gel for 15 min
    - Prepare 450 mL 1x TBE, save ~40 mL on ice for staining
    - Mix RNA with 2x gel loading buffer II; also prepare the 28/30-nt marker (from the discontinued illumina Ribo-seq kit or custom synthesized)
    - Denature at 80°C for 90 s, put on ice immediately
    - Rinse the wells using a clean transfer pipette before loading samples
    - Run the gel at 200V for 65 min
    - Stain the gel with SYBR-GOLD in ice cold 1x TBE for 3 min (4 µL SYBR-GOLD in 40 mL TBE buffer above)
    - Image using a Dark Field Transilluminator or a UV Transilluminator
    - In our experience, a clear band between 28 and 30 nt suggests good digestion for Arabidopsis

Evaluation of digestion patterns

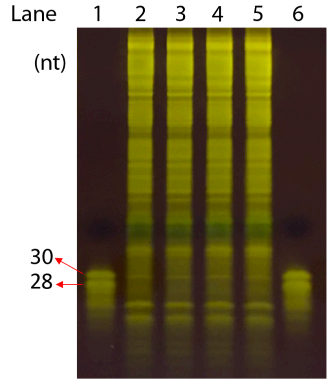

Lanes 1, 6: 28 and 30-nt marker.
Lanes 2-5: ribosome footprint samples

C. Size selection of ribosome footprints (RFs)

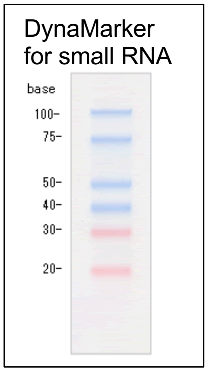

21. Prerun a 15% TBE-urea gel for 15 min
    - Prepare 400 mL 1x TBE buffer
    - Rinse the wells using a clean transfer pipette.
22. To select 20-30 nt, prepare the sRNA DynaMarker (do not heat).
23. Mix ~10 μL of RF sample from Step 18 with 10 μL of 2x gel loading buffer II.
24. Denature at 80°C for 90 s. Put on ice immediately.
25. Rinse the gel wells using a clean transfer pipette before loading the samples.
26. Separate each sample (2 wells per sample, 10 μL each) with the sRNA DynaMarker. Run at 200 V for 60 min (prepare the tubes while the gel is running).
27. Open the gel cassette and leave the gel on one side of the cassette. Put the gel and the cassette on top of a piece of white paper. Excise the gel between 20-30 nt (cut 2 wells of the same sample together) using a clean blade and place the gel slice into 1.5 mL low-bind tube.
28. Add 600 μL RNA gel extraction buffer to each sample. Make sure the gel is submerged.
29. Leave on dry ice for 30 min.
30. Thaw the samples with shaking on a nutator at room temperature overnight.
31. Briefly spin and transfer the liquid to a Ultrafree-MC GV centrifugal filter tube. Centrifuge at 2300 × g for 3 min.
32. Transfer the elution to a new 1.5-mL low-bind tube.
33. Add 2 μL of GlycoBlue and mix well.
34. Add 800 μL of isopropanol and mix well.
35. Leave the samples on dry ice for 1 hour or at -80°C overnight.
36. Centrifuge at 20,000 × g at 4°C for 30 min to pellet the RFs. (Prepare fresh 80% EtOH and leave on ice during the spin.)
37. Remove all liquid (using a 1-mL pipette first, then using a 10-μL pipette again; **DON'T spin here**).
38. Wash the pellet with 800 μL ice-cold 80% EtOH and remove the liquid without changing tips.
39. Briefly spin and remove all liquid (using a 10-μL pipette).
40. Put the tubes sideways in a microfuge tube rack. Air dry for 10 min.
41. Resuspend the RNA pellet in 3.5 μL of 10 mM Tris, pH 8.
    (Optional stopping point at -80°C)

D. Dephosphorylation and linker ligation

42. Prepare T4 PNK end-repair master mix on ice:

	Volume (µL)
T4 PNK buffer w/o ATP (10X)	0.5
T4 PNK (10 U/µL)	0.5
SUPERase-IN (20 U/µL)	0.5

43. Add 1.5 µL end-repair master mix to the 3.5 µL RF and mix.
44. Incubate at 37°C for 1 hr.
    (Optional stopping point at -80°C)
45. Prepare the linker ligation master mix on ice:

	Volume (µL)
50% w/v PEG-8000	3.5
10X T4 RNA ligase buffer	0.5
Universal miRNA cloning linker (20 µM)	0.5
T4 Rnl2(tr) K227Q (200 U/µL)	0.5

46. Add 5 µL linker ligation master mix to the 5-µL sample above and mix by pipetting.
47. Incubate at 22°C for 3 hr in a thermocycler (set the lid temperature off).
48. Deplete unligated linkers by adding:

- 0.5 µL 5'-deadenylase (10 U/µL)
- 0.5 µL RecJ exonuclease (10 U/µL)
- 30°C for 45 min

49. Purify ligations with a Zymo Oligo Clean & Concentrator kit:

- Add 39 µL of NF-H2O to each sample; now the sample volume is 50 µL.
- Proceed with the rest of the protocol.

50. Elute in 11 µL **NF-H2O**.
51. Transfer 10 µL of sample to a low-bind PCR tube.
    (Optional stopping point at -80°C)

E. Ribosomal RNA depletion

52. Thaw the following reagents for RiboZero (a component in the TruSeq Stranded Total RNA Library Prep Plant kit):

Item	Storage	Instruction
RRM-P (rRNA Removal Mix - Plant)	-80°C	Thaw, mix and put on ice
RBB (rRNA Binding Buffer)	-80°C	Thaw, mix and put on ice
RRB (rRNA Removal Beads)	4°C	**Room temperature for 30 min**

53. Save the following **<u>RNA Denaturation program</u>** in a thermocycler.
    - Preheat lid and set to 100°C
    - 68°C for 5 min
    - Hold at 4°C

54. Combine the following in a low-bind PCR tube

	1x reaction (μL)
RF from Step 51	10
RBB (buffer)	5
RPM-P	5
Total =	20

Pipet up and down 10x.

55. In a thermocycler, run the <u>RNA Denaturation program</u> (**prepare the RRB for Steps 57-58**).
56. Incubate at room temperature for 1 min.
57. Vortex RRB (beads) until well dispersed.
58. Aliquot 35 μL of RRB into each new 1.5-mL low-bind tubes.
59. Transfer the RF sample from Step 56 to the aliquoted RRB, pipette up and down 10x.
60. Incubate at room temperature for 1 min.
61. Place the reaction on a magnetic stand. Wait for 1 minute until the solution is clear.
62. Transfer the suspension (using 10-μL tips) to a new 1.5 mL low-bind tube. Expect to recover ~40 μL.
63. Purify RFs with a Zymo Oligo Clean & Concentrator kit:

- Add NF-H2O to each sample to a final volume of 50 μL.
- Proceed with the rest of the protocol.

- Elute in 11 μL 10 mM Tris pH 8. Transfer 10 μL to low-bind PCR tubes and proceed to the next step.

F. Reverse transcription and cDNA purification

64. Add 2 μL RT primer (1.25 μM) to all samples. Mix.
65. Denature at 65°C for 5 min. Put on ice immediately.
66. Set the thermocycler to 50°C.
67. Prepare the RT master mix on ice:

	Volume (μL)	Final
5X Protoscript II buffer	4	1x
dNTPs (**10 mM each**)	1	0.5 mM each
0.1M DTT	1	5 mM
SUPERase-IN (20 U/μL)	1	1 U/μL
Protoscript II (200 U/μL)	1	10 U/μL

68. Add 8 μL RT master mix to each sample and mix. Now the volume is 20 μL.
69. Incubate at 50°C for 30 min.
70. Add 2.2 μL of **1 M NaOH** to each reaction and mix; incubate at 70°C for 20 min.
71. Purify cDNA with a Zymo Oligo Clean & Concentrator kit:

- Add NF-H2O to each sample to a final volume of 50 μL.
- Proceed with the rest of the protocol.
- Elute in 6.5 μL 10 mM Tris pH8. Transfer to low-bind PCR tubes.

72. Prerun a 10% TBE-urea gel at 200 V for 15 min in 1x TBE.
    - Prepare 450 mL 1x TBE buffer.
    - **Save 40 mL of 1x TBE buffer and leave on ice for gel staining.**
    - Rinse the wells using a clean transfer pipette.
73. Add 6.5 μL of 2x gel loading buffer II to each sample. Also prepare the 20/100 ssDNA ladder needed to separate each sample.
74. Denature the samples and ladder at 80°C for 90 s. Move to ice immediately.
75. Thoroughly rinse the gel wells, then load the samples and ladder onto a denaturing 10% TBE-urea gel.
76. Run at 200 V for 80 min in 1× TBE buffer (**put the 40 mL 1x TBE on ice**).

77. Stain the gel with SYBR-GOLD in ice-cold 1x TBE for 3 min (4 µL SYBR-GOLD in 40 mL TBE buffer above).
78. Visualize gel using a Dark Field Transilluminator. Excise the RT products between 94 and 104 nt using a clean blade (do not cut a wider range; it will increase unwanted PCR products). Place the excised gel in a 1.5-mL low-bind tube.
79. Add 500 µL **DNA** extraction buffer. Extract the cDNA as described in Steps 28-39.
80. Resuspend the cDNA in 15 µL 10 mM Tris pH 8 and transfer to a low-bind PCR tube.
    (Optional stopping point at -20°C overnight or -80°C for longer periods)

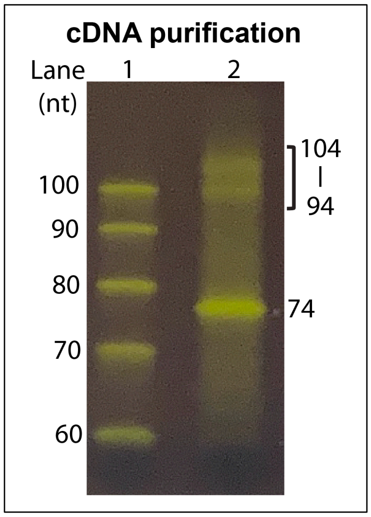

G. Circularization of cDNA

81. Prepare circularization master mix on ice:

	Volume (µL)	Final
10x CircLigase buffer	2	1x
1 mM ATP	1	50 µM
50 mM MnCl2	1	2.5 mM
CircLigase (100 U/µL)	1	100U

82. Add 5 µL of circularization master mix to each sample and mix. Now the sample volume is 20 µL.
83. Incubate with the following program in a thermocycler:
    60°C – 2 hr
    80°C – 10 min
    4°C – hold.
    (Optional stopping point: store at -20°C)

H. qPCR quantification of circularized cDNA

84. Prepare a dilution series of the positive control:
    - Mix 2 µL of a **1 µM** stock with 198 µL NF-H2O (10 nM).
    - Mix 10.2 µL of the 10 nM solution above with 89.8 µL of NF-H2O (1.02 nM).
    - Serially dilute 3 µL of the stock into 9 µL of NF-H2O to prepare a 1:4 dilution (~256 pM), a 1:16 dilution (64 pM), a 1:64 dilution (16 pM), a 1:256 dilution (4 pM), and a 1:1024 dilution (1 pM).
    - **Also prepare 0.5 nM negative control (reverse transcription primer).**
85. Mix 1 µL of circularized cDNA from Step 83 with 9 µL NF-H2O.
86. Set up qPCR as follows:
    - Serial dilutions and controls (total of 8): 1.02 nM, 256 pM, 64 pM, 16 pM, 4 pM, 1 pM, NF-H2O, negative control.
    - Each circularization requires 2 technical replicates.
87. Prepare qPCR master mix on ice:

	1 reaction	________ reactions	Final
NF-H2O	6.4		
2x Luna qPCR master mix	10		1X
10 µM primer F	0.8		0.4 µM
10 µM primer R	0.8		0.4 µM

88. For each reaction, combine 18 µL of qPCR master mix with 2 µL of template, pipette 10 times.
89. Spin down and perform qPCR amplification using the following cycling conditions:
    - 95° C, 60 s
    - Repeat 40 cycles of
      95 °C, 15 s
      59 °C, 30 s
    - Melting curve analysis.

90. Fit a standard curve to the Cq values for the serial dilution series. The 1.02 nM sample should have a Cq of roughly 8. Verify that the negative control and the blank reactions have Cq values much higher than the standard curve or circularized cDNA samples.
91. Determine the template concentration in the circularization reactions based on the standard curve.

92. Select the amount of template and the number of cycles for the library construction PCR based on the table below for a 50-µL PCR reaction.

Template concentration	Cycles needed
800 pM	7
400 pM	8
200 pM	9
100 pM	10
50 pM	11
25 pM	12
12.5 pM	13
6.25 pM	14
3.125 pM	15
1.6 pM	16

93. If a template is at 700 pM, 2.3 µL of template will have a concentration of 32 pM in a 50 µL reaction, so ~12 cycles will be needed.
    - Make sure the template comprises no more than 10% of the final PCR volume and ideally no more than 5%. This helps minimize reannealed duplexes.

I. Library construction PCR

94. For each sample, set up a 50-µL PCR reaction on ice:

	Volume (µL)
NF-H2O	20-X
2X Phusion HF master mix	25
10 µM Forward library PCR primer	2.5
10 µM Reverse ***INDEXED*** primer	2.5
Circularized cDNA	X

95. Purify the PCR products using a DNA Clean & Concentrator 5 kit. Elute in 25.5 µL NF-H2O (recover ~25 µL).
96. Add 5 µL of 6x DNA gel loading dye (purple) to each sample.
97. Prepare the 20-bp ladder.

98. Set up a pre-cast 8% polyacrylamide non-denaturing gel (prepare 450 mL 1x TBE and save 40 mL on ice for staining).

99. Load 3 adjacent wells with 10 µL each of the purified PCR samples; separate different samples with 3 µL of 20 bp ladder.

100. Run the gel at 200 V for 40 min in 1X TBE.

101. Stain the gel with ice-cold 1x TBE/SYBR Gold for 3 min (4 µL SYBR-GOLD in 40 mL TBE buffer above)

102. Excise the expected library band:
   - For 20-30-nt footprints, the library should be **162-172 bp**.
   - Avoid bands that are ~145 bp and below (these are products resulting from self-ligated universal miRNA cloning linker and self-circularized reverse transcription primer).

103. Extract DNA as described in Step 79.

104. Resuspend the DNA in 11 µL of 10 mM Tris pH 8.

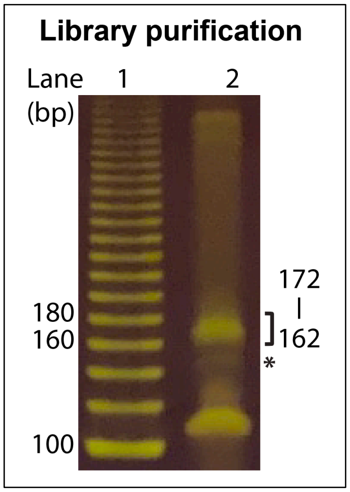

J. Library QC and pooling

105. Quantify the library DNA with a Qubit DNA HS assay.

106. Analyze library size distributions with Agilent Fragment Analyzer. Libraries should be roughly 170 bp.

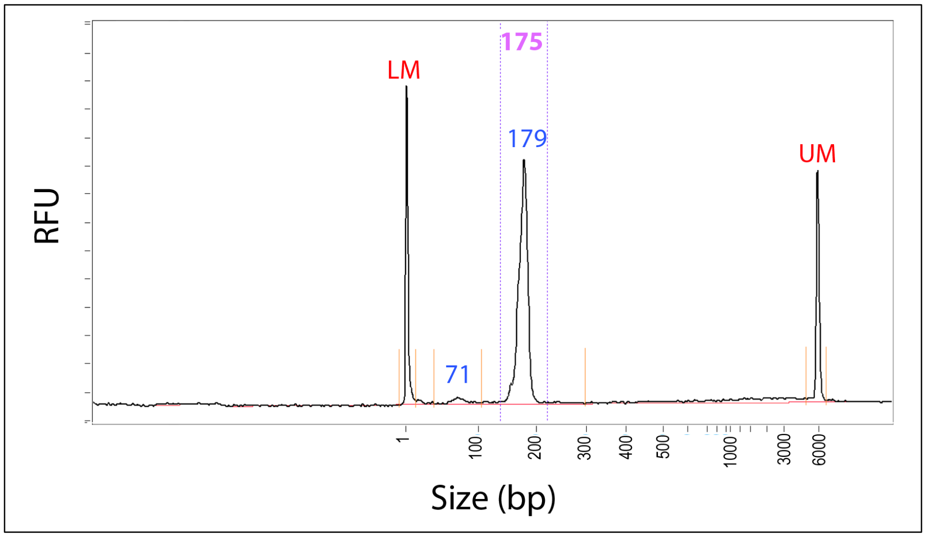

107. Pool libraries with the same molarity.

108. Proceed with single-end 50-bp sequencing.
