# Supplementary material for: A custom library construction method for super-resolution ribosome profiling in Arabidopsis
Source: Plant Methods. 2022 Oct 4;18:115. doi: 10.1186/s13007-022-00947-2 (PMC9531494; doi:10.1186/s13007-022-00947-2)
Supplement: Supplementary file 4 — Additional file 4. Markdown file of example data analysis. [file 13007_2022_947_MOESM4_ESM.pdf]

ADDITIONAL FILE 4
Description:

1. This github repository is for the **A custom library construction method for super-resolution ribosome profiling in Arabidopsis** paper currently on biorxiv. Although this paper is about how to create Ribo-seq library, we provide a simple pipeline for the quality control here. Please cite this paper if you find the information provided here is useful.
2. You can run the code on locally (PC/Mac), linux server or linux cluster with properly installed/loaded software packages.
3. You have to decide the $INPUT and $OUTPUT paths for each step.
4. Here we provide a small example from the sample NEB1. The FASTA and transcriptome GTF files can be obtained from **TAIR**. Use Chrome browser. Connect as a guest.
5. This is beyond the scope of the paper, but more info about downstream analysis of Riboseq can be found **HERE**. For visualizing the Ribo-seq reads on individual transcripts, please try out **RiboPlotR**.
6. If run with > R (v. 4.2), there is a known issue for RiboseQC (from the GenomicFeatures package). A modified version of RiboseQC is available **HERE**. Use `install_github(repo = "hsinyenwu/RiboseQC_R4.2.1")` then load the package with `library(RiboseQC)`.

Prepare example file from the sequencing file

The NEB1.fastq.gz is available on NCBI SRA BioProject ID PRJNA854638 after the project is published. We selected the first 125000 reads as the example.

```
zcat NEB1.fastq.gz | head -500000 > NEB1.halfM.fastq.gz #Extract first 125000 reads
i.e., 0.5 million lines, 4 lines of a fasta is one sequencing read.
```

Step 1 code (FASTQC for quality check):

```
#$OUTPUT output directory
fastqc -o $OUTPUT -t 10 NEB1.halfM.fastq.gz
```

Step 2 code (FASTX toolkit to trim adaptor sequence):

```
#$INPUT input directory
#$OUTPUT output directory
#Please see FASTX toolkit online page for the parameters used here
zcat $INPUT/NEB1.halfM.fastq.gz | fastx_clipper -o $OUTPUT/NEB1.halfM.trim.fastq -a
CTGTAGGCACCATCAAT -l 20 -c -n -v
```

Step 3a code (Bowtie2: Build index for contamination sequences)

```
#$CF is the directory contain FASTA files for contamination sequence
#$OUTPUT output directory
cd $OUTPUT
bowtie2-build $CF/Araport11_contam5.fa Contam5
```

Step 3b code (Bowtie2: remove contamination sequences)

```
#$Contam5 is the directory with the index for the contamination sequences
bowtie2 -L 20 -p 8 -x $Contam5 $INPUT/NEB1.halfM.trim.fastq --un-gz
$OUTPUT/NEB1.halfM.trim.noContam5.fastq.gz
```

Step 4a code (STAR: create genome/transcriptome index for STAR)

```
#$starIndex is the directory contain FASTA files
#$FASTA is the path to the TAIR10 genome FASTA file
#$GTF is the path to the Araport11 GTF file
#$OUTPUT is the output directory
#Please see STAR aligner online page for the parameters used here

cd $OUTPUT
STAR --runThreadN 12 \
--runMode genomeGenerate \
--genomeDir $starIndex \
--genomeFastaFiles $FASTA \
--sjdbGTFfile $GTF \
--sjdbOverhang 34 \
```

Step 4b code (STAR mapping)

```
#$starIndex is the directory contain index files
#$INPUT is the directory with the NEB1.halfM.trim.noContam5.fastq.gz file
#$OUTPUT is the output directory
#Please see STAR aligner online page for the parameters used here

STAR --runThreadN 10 \
--genomeDir $starIndex \
--readFilesCommand zcat \
--readFilesIn $INPUT/NEB1.halfM.trim.noContam5.fastq.gz \
--alignIntronMax 5000 \
--alignIntronMin 15 \
```

```
--outFilterMismatchNmax 1 \
--outFilterMultimapNmax 20 \
--outFilterType BySJout \
--alignSJoverhangMin 8 \
--alignSJDBoverhangMin 2 \
--outSAMtype BAM SortedByCoordinate \
--quantMode TranscriptomeSAM \
--outSAMmultNmax 1 \
--outMultimapperOrder Random \
--outFileNamePrefix "star_ribo_NEB1_halfM" \
```

Step 5 code (RiboseqQC: qulaity control for ribo-seq data)

```
#Create 2bit files
#Run the code in R (v. 4.1.3)
#Please set the proper path for required files in your system (e.g., if you run on
mac, it could be ~/Desktop/Ribo_example)

library(Biostrings)
library(rtracklayer)
library(RiboseQC)
#If run with > R (v. 4.2), there is a known issue for RiboseQC (from the
GenomicFeatures package). A modified version of RiboseQC is available [here]
(https://github.com/hsinyenwu/RiboseQC_R4.2.1/blob/master/R/riboseqc.R). Use
```install_github(repo = "hsinyenwu/RiboseQC_R4.2.1")``` then load the package with
```library(RiboseQC)```.

At_genome_seqs <-
Biostrings::readDNAStringSet("~/Desktop/Ribo_example/TAIR10_chr_all_2.fas")
At_genome_seqs <- replaceAmbiguities(At_genome_seqs)
test_2bit_out <- file.path("~/Desktop/Ribo_example", "At.2bit")
rtracklayer::export.2bit(At_genome_seqs, test_2bit_out)

setwd("$PATH")

prepare_annotation_files(annotation_directory = ".",
                         twobit_file = "/At.2bit",
                         gtf_file =
"~/Desktop/Ribo_example/Araport11_20220629.gtf",scientific_name =
"Arabidopsis.thaliana",
                         annotation_name = "TAIR10",export_bed_tables_TxDb =
F,forge_BSgenome = T,create_TxDb = T)

RiboseQC_analysis(annotation_file="~/Desktop/Ribo_example/Araport11_20220629.gtf_Rannot
 =
"~/Desktop/Ribo_example/star_ribo_NEB1_halfM_Aligned.sortedByCoord.out.bam",report_file
 = "star_ribo_NEB1_halfM_rm_Contam5.html",write_tmp_files = F)
```

Example gtf file (the 9th column is the key for running RiboseQC):

```
1   Araport11   gene    3631    5899    .   +   .   gene_id "AT1G01010"; gene_biotype "protein_coding";
1   Araport11   CDS 3760    3913    .   +   0   gene_id "AT1G01010"; transcript_id "AT1G01010.1"; gene_biotype "protein_coding";
1   Araport11   CDS 3996    4276    .   +   2   gene_id "AT1G01010"; transcript_id "AT1G01010.1"; gene_biotype "protein_coding";
1   Araport11   CDS 4486    4605    .   +   0   gene_id "AT1G01010"; transcript_id "AT1G01010.1"; gene_biotype "protein_coding";
1   Araport11   CDS 4706    5095    .   +   0   gene_id "AT1G01010"; transcript_id "AT1G01010.1"; gene_biotype "protein_coding";
1   Araport11   CDS 5174    5326    .   +   0   gene_id "AT1G01010"; transcript_id "AT1G01010.1"; gene_biotype "protein_coding";
1   Araport11   CDS 5439    5630    .   +   0   gene_id "AT1G01010"; transcript_id "AT1G01010.1"; gene_biotype "protein_coding";
1   Araport11   transcript  3631    5899    .   +   .   gene_id "AT1G01010"; transcript_id "AT1G01010.1"; gene_biotype "protein_coding";
1   Araport11   exon    3631    3913    .   +   .   gene_id "AT1G01010"; transcript_id "AT1G01010.1"; gene_biotype "protein_coding";
1   Araport11   exon    3996    4276    .   +   .   gene_id "AT1G01010"; transcript_id "AT1G01010.1"; gene_biotype "protein_coding";
1   Araport11   exon    4486    4605    .   +   .   gene_id "AT1G01010"; transcript_id "AT1G01010.1"; gene_biotype "protein_coding";
1   Araport11   exon    4706    5095    .   +   .   gene_id "AT1G01010"; transcript_id "AT1G01010.1"; gene_biotype "protein_coding";
1   Araport11   exon    5174    5326    .   +   .   gene_id "AT1G01010"; transcript_id "AT1G01010.1"; gene_biotype "protein_coding";
1   Araport11   exon    5439    5899    .   +   .   gene_id "AT1G01010"; transcript_id "AT1G01010.1"; gene_biotype "protein_coding";
1   Araport11   five_prime_UTR  3631    3759    .   +   .   gene_id "AT1G01010"; transcript_id "AT1G01010.1"; gene_biotype "protein_coding";
1   Araport11   three_prime_UTR 5631    5899    .   +   .   gene_id "AT1G01010"; transcript_id "AT1G01010.1"; gene_biotype "protein_coding";
1   Araport11   gene    163278  166353  .   +   .   gene_id "AT1G01448"; gene_biotype "antisense_long_noncoding_rna";
1   Araport11   transcript  163278  166353  .   +   .   gene_id "AT1G01448"; transcript_id "AT1G01448.1"; gene_biotype "antisense_long_noncoding_rna";
1   Araport11   exon    163278  163516  .   +   .   gene_id "AT1G01448"; transcript_id "AT1G01448.1"; gene_biotype "antisense_long_noncoding_rna";
1   Araport11   exon    163934  164103  .   +   .   gene_id "AT1G01448"; transcript_id "AT1G01448.1"; gene_biotype "antisense_long_noncoding_rna";
1   Araport11   exon    164225  164686  .   +   .   gene_id "AT1G01448"; transcript_id "AT1G01448.1"; gene_biotype "antisense_long_noncoding_rna";
1   Araport11   exon    164771  165380  .   +   .   gene_id "AT1G01448"; transcript_id "AT1G01448.1"; gene_biotype "antisense_long_noncoding_rna";
```

```
1   Araport11   exon    165449  166353  .   +   .   gene_id "AT1G01448";
transcript_id "AT1G01448.1"; gene_biotype "antisense_long_noncoding_rna";
1   Araport11   transcript  163278  166353  .   +   .   gene_id "AT1G01448";
transcript_id "AT1G01448.2"; gene_biotype "antisense_long_noncoding_rna";
1   Araport11   exon    163278  163516  .   +   .   gene_id "AT1G01448";
transcript_id "AT1G01448.2"; gene_biotype "antisense_long_noncoding_rna";
1   Araport11   exon    163934  164103  .   +   .   gene_id "AT1G01448";
transcript_id "AT1G01448.2"; gene_biotype "antisense_long_noncoding_rna";
1   Araport11   exon    164225  164686  .   +   .   gene_id "AT1G01448";
transcript_id "AT1G01448.2"; gene_biotype "antisense_long_noncoding_rna";
1   Araport11   exon    164771  165380  .   +   .   gene_id "AT1G01448";
transcript_id "AT1G01448.2"; gene_biotype "antisense_long_noncoding_rna";
1   Araport11   exon    165449  165656  .   +   .   gene_id "AT1G01448";
transcript_id "AT1G01448.2"; gene_biotype "antisense_long_noncoding_rna";
1   Araport11   exon    165769  166353  .   +   .   gene_id "AT1G01448";
transcript_id "AT1G01448.2"; gene_biotype "antisense_long_noncoding_rna";
1   Araport11   transcript  163302  166307  .   +   .   gene_id "AT1G01448";
transcript_id "AT1G01448.3"; gene_biotype "antisense_long_noncoding_rna";
1   Araport11   exon    163302  163516  .   +   .   gene_id "AT1G01448";
transcript_id "AT1G01448.3"; gene_biotype "antisense_long_noncoding_rna";
1   Araport11   exon    163934  164103  .   +   .   gene_id "AT1G01448";
transcript_id "AT1G01448.3"; gene_biotype "antisense_long_noncoding_rna";
1   Araport11   exon    164225  164686  .   +   .   gene_id "AT1G01448";
transcript_id "AT1G01448.3"; gene_biotype "antisense_long_noncoding_rna";
1   Araport11   exon    164771  165380  .   +   .   gene_id "AT1G01448";
transcript_id "AT1G01448.3"; gene_biotype "antisense_long_noncoding_rna";
1   Araport11   exon    165449  165656  .   +   .   gene_id "AT1G01448";
transcript_id "AT1G01448.3"; gene_biotype "antisense_long_noncoding_rna";
1   Araport11   exon    165983  166307  .   +   .   gene_id "AT1G01448";
transcript_id "AT1G01448.3"; gene_biotype "antisense_long_noncoding_rna";
Pt  Araport11   gene    60741   61430   .   +   .   gene_id "ATCG00530";
gene_biotype "protein_coding";
Pt  Araport11   CDS 60741   61430   .   +   0   gene_id "ATCG00530"; transcript_id
"ATCG00530.1"; gene_biotype "protein_coding";
Pt  Araport11   transcript  60741   61430   .   +   .   gene_id "ATCG00530";
transcript_id "ATCG00530.1"; gene_biotype "protein_coding";
Pt  Araport11   exon    60741   61430   .   +   .   gene_id "ATCG00530";
transcript_id "ATCG00530.1"; gene_biotype "protein_coding";
Mt  Araport11   gene    366086  366700  .   -   .   gene_id "ATMG01410";
gene_biotype "protein_coding";
Mt  Araport11   CDS 366086  366700  .   -   0   gene_id "ATMG01410"; transcript_id
"ATMG01410.1"; gene_biotype "protein_coding";
Mt  Araport11   transcript  366086  366700  .   -   .   gene_id "ATMG01410";
transcript_id "ATMG01410.1"; gene_biotype "protein_coding";
Mt  Araport11   exon    366086  366700  .   -   .   gene_id "ATMG01410";
transcript_id "ATMG01410.1"; gene_biotype "protein_coding";
```

Note:

Below the examples require all data (i.e., NEB1,NEB2,NEB3. Not only the small example file). Please download the files from SRA after the paper is published.

Step 6a: Kallisto (v0.46.1) indexing:

```
#$OUTPUT is the path to the output folder
#$FASTA is the path to a fasta file with only the CDS regions (not the genome or
transcriptome fasta)
kallisto index -i $OUTPUT/transcripts.idx $FASTA -k 19
```

Step 6b: Kallisto mapping for Ribo-seq reads

```
kallisto quant -i $Index/transcripts.idx -o $OUTPUT1 -t 10 --single -l 28 -s 2
$INPUT/NEB1.noContam5.fastq.gz
kallisto quant -i $Index/transcripts.idx -o $OUTPUT2 -t 10 --single -l 28 -s 2
$INPUT/NEB2.noContam5.fastq.gz
kallisto quant -i $Index/transcripts.idx -o $OUTPUT3 -t 10 --single -l 28 -s 2
$INPUT/NEB3.noContam5.fastq.gz
```

Step 6c: Correlation plot with R

```
library(dplyr)
library(corrplot)
RiboD1 <-
read.delim("~/Desktop/New_Riboseq/NEB1_abundance.tsv",header=T,sep="\t",stringsAsFactor
 = F,quote = "")
RiboD2 <-
read.delim("~/Desktop/New_Riboseq/NEB2_abundance.tsv",header=T,sep="\t",stringsAsFactor
 = F,quote = "")
RiboD3 <-
read.delim("~/Desktop/New_Riboseq/NEB3_abundance.tsv",header=T,sep="\t",stringsAsFactor
 = F,quote = "")

TPM=data.frame(RiboD1=RiboD1$tpm,RiboD2=RiboD2$tpm,RiboD3=RiboD3$tpm)

TPM$Ribo_mean <-rowMeans(TPM[,1:3])

TPM2 <- TPM %>% filter(Ribo_mean>0.1) %>% filter(Ribo_mean<200)

R2 <- round(cor(TPM2[,1:3]),2)
```

```
pdf("~/Desktop/New_Riboseq/NEB_samples_correlation.pdf",width =4,height = 4)
corrplot(R2, method = "number",type="upper")
dev.off()
```

Step 7: Calculate 3-nt periodicity with R (can run with the example file)

```
# Load the results_RiboseQC file from RiboseQC output
load("~/Desktop/New_Riboseq/star_ribo_NEB1_halfM_Aligned.sortedByCoord.out.bam_results_

# Extract P_sites_subcodon
P_sites_subcodon_readCount = res_all[["profiles_P_sites"]][["P_sites_subcodon"]]
[["nucl"]][["28"]]
# Print out P_sites_subcodon_readCount
P_sites_subcodon_readCount

# Get the CDS region
P_CDS = P_sites_subcodon_readCount[51:149] #93 nt total, 33 nt start and after, 33
nt in the middle, 27 nt from -2 codon of stop codon to -11 codon of stop codon

# Get total number of reads for 3 frames
F1 = sum(P_CDS[seq(1,93,by=3)])
F2 = sum(P_CDS[seq(2,93,by=3)])
F3 = sum(P_CDS[seq(3,93,by=3)])
# Calculate 3-nt periodicity
F1/(F1+F2+F3)*100
```
