# Supplementary material for: A custom library construction method for super-resolution ribosome profiling in Arabidopsis
Source: Plant Methods. 2022 Oct 4;18:115. doi: 10.1186/s13007-022-00947-2 (PMC9531494; doi:10.1186/s13007-022-00947-2)
Supplement: Supplementary file 5 — Additional file 5: Figure S1. Precise ribonuclease digestion yields a clear band between 28 and 30 nt. Figure S2. qPCR quantification of circularized cDNAs and estimation of template volumes needed for library construction PCR. Figure S3. Comparison of rRNA contamination in our previous and current datasets. Figure S4. High correlations among three technical replicates of Ribo-seq data. Figure S5. Comparable strong 3-nt periodicity in our current data. Figure S6. Abundant contamination sequences present at 25 nt. Figure S7. Mapping and contamination statistics. Contaminant sequences considered include rRNAs/tRNAs/snRNAs/snoRNAs and overrepresented non-coding RNAs. Their sequences are listed in Additional file 3. [file 13007_2022_947_MOESM5_ESM.pdf]

ADDITIONAL FILE 5

Figure S1

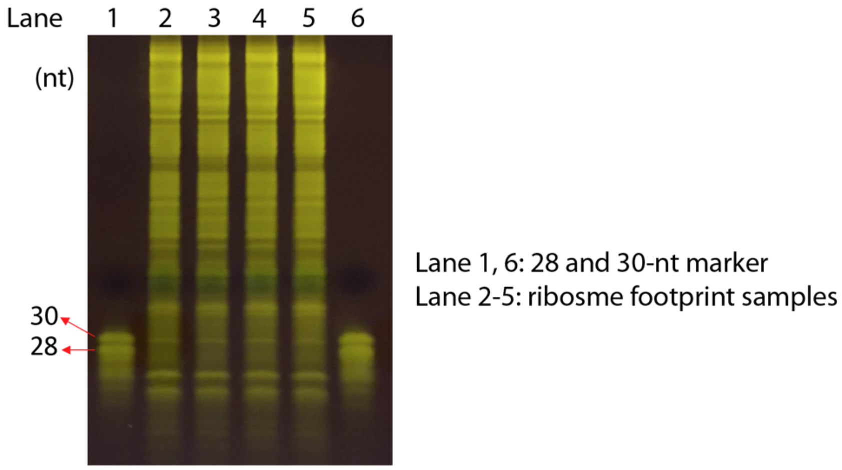

Figure S1. Precise ribonuclease digestion yields a clear band between 28-30 nt. About 200-ng RNA (after monosome isolation) was separated by 15% TBE-Urea gel to evaluate the digestion patterns.

Figure S2

A.

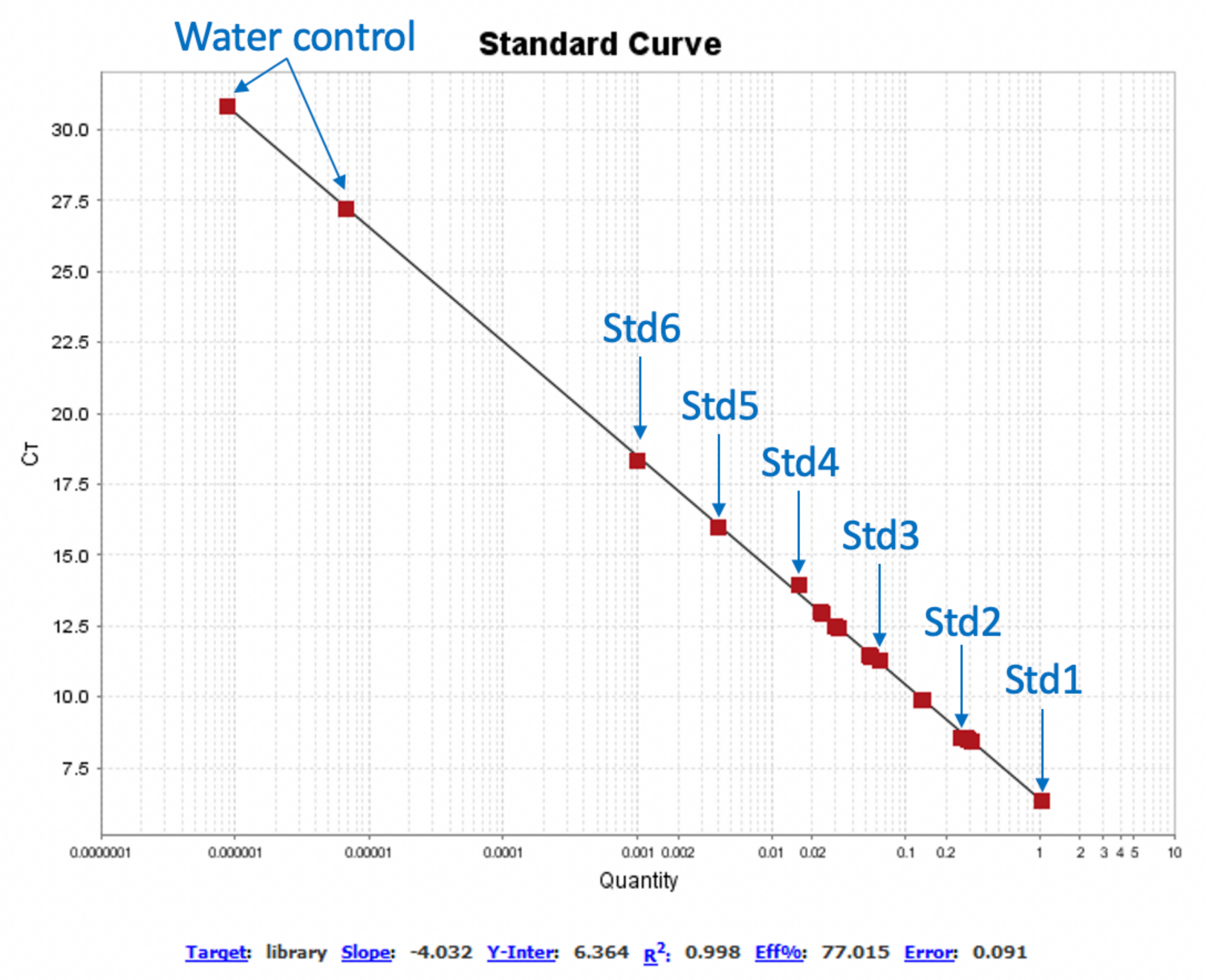

B.

	Sample 10x dilution (nM)	Sample pM	µl template in 50 µl PCR
Sample 1	0.1341	1341	1.9
Sample 2	0.2995	2995	0.8
Sample 3	0.0548	548	4.6

Figure S2. qPCR quantification of circularized cDNAs and estimation of template volumes needed for library construction PCR.

(A) The standard curve of the qPCR quantification. A serial dilution of a synthetic oligo (its sequence was listed in File S1) was set up as previously described [1] to establish the

standard curve. Standard 1 (Std1) is 1.024 nM, Std2 is 0.256 nM, Std3= 0.064 nM, and so on. This dilution series covers our sample concentrations well. The three samples (10-fold dilution) were quantified as 0.1341, 0.2995, and 0.0548 nM (highlighted in red in B)

(B) Calculation of the template volumes needed for library PCR. According to the template concentration and PCR cycles recommended by [1], PCR of 11 cycles requires a template concentration 50 pM in 50 µL PCR reaction. Thus, template volume (µL) needed = (50 pM x 50 µL) / Sample concentration (pM). As recommended, 1.9, 0.8 and 4.6 µL of cDNA for the three samples were used for library PCR.

Figure S3. **Comparison of rRNA contamination in our previous and current datasets.**

Dataset	Library	Total reads	rRNA reads	rRNA %
**Hsu *et al.* 2016 [2]**	Root 1	157071952	63281561	40.3%
	Root 2	151506822	50087179	33.1%
	Root 3	162527177	67669744	41.6%
	Shoot 1	143249429	27357517	19.1%
	Shoot 2	177659312	49638803	27.9%
	Shoot 3	128122140	46114428	36.0%
**Current study**	Seedling 1	49917617	14643119	29.3%
	Seedling 2	51209491	14690021	28.7%
	Seedling 3	41968248	12058118	28.7%

Figure S4

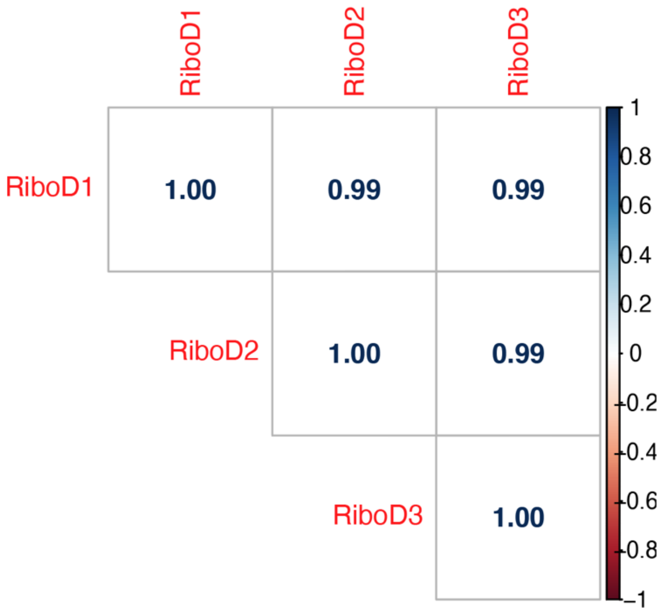

Figure S4. High correlations among three technical replicates of Ribo-seq data. Ribo-seq reads mapped to individual transcripts (considering transcript per million, TPM) were compared among the three technical replicates. Pearson correlations between the samples are shown.

Figure S5

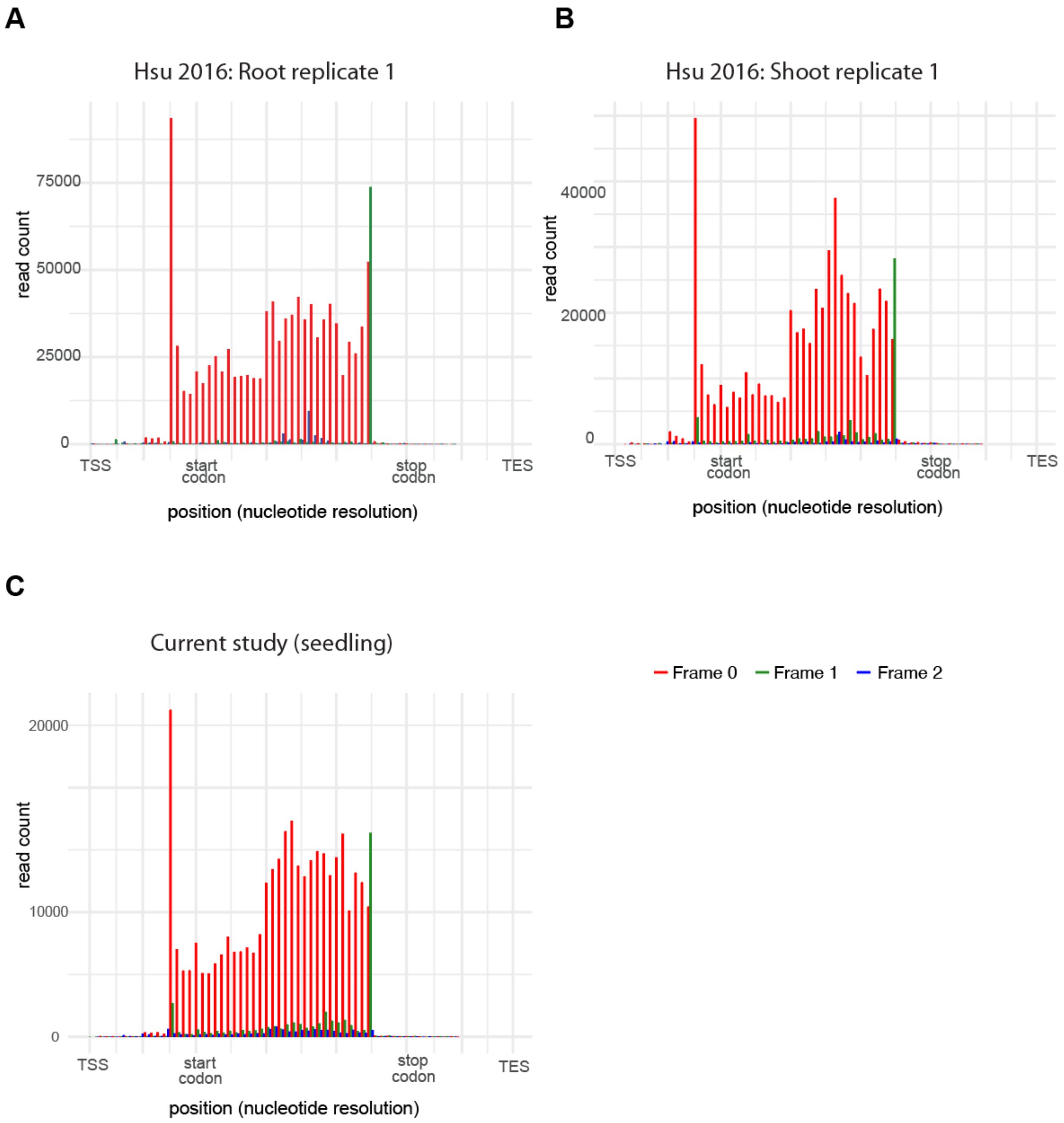

Figure S5. Comparable strong 3-nt periodicity in our current data. Metaplots (global analysis of Ribo-seq reads) of our previous data in Arabidopsis root (A) and shoot (B) and current study (C). The expected reading frame (Frame 0, according to the annotation) is shown in red, the other two reading frames are shown in green and blue.

Figure S6

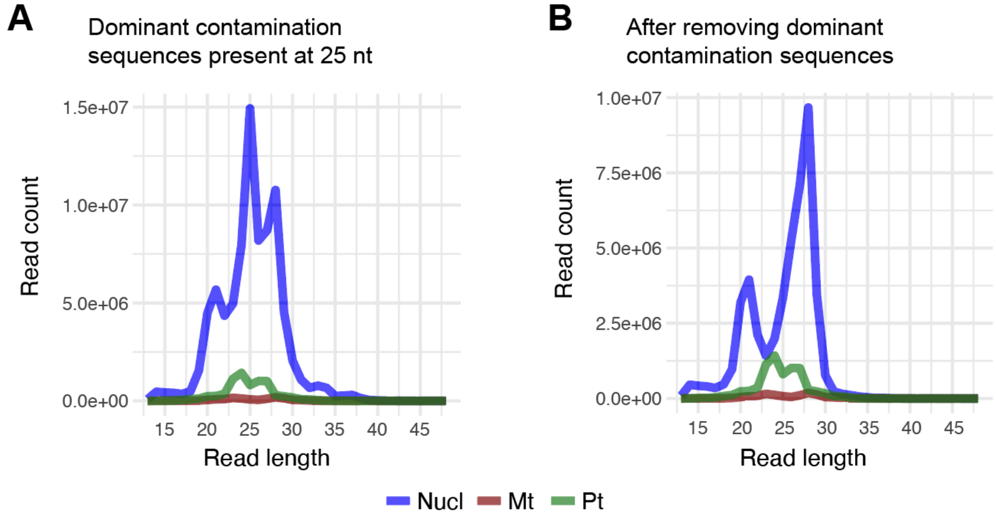

Figure S6. Abundant contamination sequences present at 25 nt. (A) Read length distribution after removing common contaminants from rRNAs, tRNAs, snRNAs and snoRNAs. A major peak at 25 nt mainly mapped to non-coding RNAs *AT3G06365* and *AT2G03875*. (B) Read length distribution after removing the additional contaminants from *AT3G06365* and *AT2G03875*.

Figure S7. Mapping and contamination statistics. Contaminant sequences considered include rRNAs/tRNAs/snRNAs/snoRNAs and overrepresented non-coding RNAs. Their sequences are listed in File S3.

	Total reads (M)	Contam. (M)	Contam. %	Non-contam. (M)	Non-contam. %	Mapped reads (M)	Mapped % in non-contam.	Mapped % in total reads
Sample-1	49.9	29.8	59.8	20.1	40.2	12.7	63.4	25.5
Sample-2	51.2	30.2	58.9	21.0	41.1	12.9	61.5	25.2
Sample-3	42.0	23.6	56.2	18.4	43.8	11.4	62.1	27.2

Reference:

1. McGlincy NJ, Ingolia NT. Transcriptome-wide measurement of translation by ribosome profiling. Methods. 2017;126:112–29.

2. Hsu PY, Calviello L, Wu H-YL, Li F-W, Rothfels CJ, Ohler U, et al. Super-resolution ribosome profiling reveals unannotated translation events in Arabidopsis. Proceedings of the National Academy of Sciences of the United States of America. 2016;113:E7126–35.
